# Considerable doubt about rubella screening and vaccination among unvaccinated orthodox protestant women: a mixed-methods study

**DOI:** 10.1186/s12889-023-15625-8

**Published:** 2023-04-14

**Authors:** Anne C. de Munter, Jeannine L. A. Hautvast, Wilhelmina L. M. Ruijs, Robert A. C. Ruiter, Marlies E. J. L. Hulscher

**Affiliations:** 1 Radboud Institute for Health Sciences, Department of Primary and Community Care, Radboud university medical center, Postbus, Nijmegen, 9101 6500 HB The Netherlands; 2 Department of Infectious Disease Control, GGD Gelderland-Zuid, Postbus, Nijmegen, 1120, 6501 BC The Netherlands; 3 Department of Health Care, GGD GHOR Nederland, Zwarte Woud 2, Utrecht, 3524 SJ The Netherlands; 4 National Institute for Public Health and the Environment, Centre for Infectious Disease Control, PO box 1, Bilthoven, 3720 BA The Netherlands; 5 Department of Work & Social Psychology, Maastricht University, PO Box 616, Maastricht, 6200 MD The Netherlands; 6 Radboud university medical center, Radboud Institute for Health Sciences, IQ healthcare, Postbus, Nijmegen, 9101, 6500 HB The Netherlands

**Keywords:** Decision making, Intention, Vaccine hesitancy, Rubella, Religious belief

## Abstract

**Background:**

Women who are susceptible to rubella are advised to vaccinate against rubella to prevent infection in future pregnancies, and thus avert the risk of congenital rubella syndrome in their unborn child. Rubella outbreaks periodically occur in the under-vaccinated orthodox Protestant community in the Netherlands. The objective of this mixed-methods study was to determine and understand personal experience with rubella, perceived rubella susceptibility, and intention to accept rubella screening and vaccination among unvaccinated orthodox Protestant women. The ultimate aim of this study was to inform policy and practice and contribute to the prevention of cases of congenital rubella syndrome.

**Methods:**

A mixed-methods study was conducted combining an online survey and semi-structured interviews among unvaccinated Dutch orthodox Protestant women aged 18–40 years. Descriptive analysis was used for quantitative data. Qualitative data was analysed using codes and categories.

**Results:**

Results of the survey (167 participants) showed that most participants had personal experience with rubella (74%, 123/167) and 101 women (61%, 101/167) indicated they had had rubella themselves. More than half of the women were undecided whether to accept rubella susceptibility screening (56%; 87/156) or rubella vaccination (55%; 80/146). Qualitative findings (10 participants) showed that most women thought they were not susceptible to rubella. Indecisiveness and negative attitudes to accept rubella vaccination were related with religious arguments to object vaccination and with women’s perception of absence of imminent threat of rubella. Furthermore, results showed presence of misconceptions among women in the interpretation of their susceptibility and high confidence in their parents’ memory that they had experienced rubella as a child although no laboratory screening had been conducted.

**Conclusions:**

In light of an imminent rubella outbreak in the Netherlands, a tailored education campaign should be prepared aimed at and established in cooperation with the under-vaccinated orthodox Protestant community. Health care providers should provide adequate information on rubella and support decision-making in order to stimulate women to make a deliberate and informed decision on rubella screening and, if necessary, subsequent vaccination.

## Introduction

Rubella is a highly contagious, yet, generally non-severe disease that passes with mild or no symptoms [1]. However, rubella infection during pregnancy, particularly in the first trimester, can result in miscarriage and stillbirth, and/or cause severe complications in the developing fetus, known as congenital rubella syndrome (CRS). CRS is characterised by ophthalmological, cardiac, brain, genitourinary and other abnormalities, including hearing loss and low birth weight[2]. Many countries offer rubella vaccination to all children in vaccination programmes, often in a combination vaccine, e.g. measles-mumps-rubella (MMR-)vaccination [3, 4]. In the Netherlands, children are offered two MMR-vaccinations at the ages of 14 months (MMR1), and 9 years (MMR2) [5]. Both vaccination and natural infection provide lifelong immunity against rubella [4].

To eliminate rubella, countries need a high vaccination uptake. In countries with a near-optimal vaccination coverage, the incidence of rubella is reduced, however, the disease is not eliminated. In these countries, longer time periods between rubella outbreaks may be observed, increasing the average age of infection and making it an adult disease as well [6]. For this reason, children are born with CRS in countries with successful vaccination programmes. Despite the goal to eliminate rubella in the WHO European region, 27 CRS cases have been reported in Italy, and seven in both Spain and Portugal in the last 15 years [7].

CRS can be prevented by providing rubella vaccination to susceptible women of childbearing age. Since women who are still susceptible to rubella are rarely registered as such in a national registration system, rubella susceptibility screening is offered to pregnant women during antenatal care [8]. As the live attenuated rubella vaccine is contraindicated during pregnancy, vaccination to susceptible women can only be provided after pregnancy. Many European countries that provide rubella susceptibility screening programmes target all pregnant women, independent of their immunity status [8]. In the Netherlands, national guidelines advise health care providers (HCPs), e.g. midwives, gynaecologists and general practitioners, to offer screening to unvaccinated women and women with an unknown vaccination status during pregnancy [9, 10].

In 2021, the Netherlands had a first dose MMR-vaccination coverage of 93% among young children, and a second dose MMR-vaccination coverage of 90% among adolescents [11]. Part of those who are not MMR vaccinated during childhood, belong to the orthodox Protestant minority; a socially and geographically clustered close-knit community with low vaccination coverage due to religious objections [12–14]. Today, the orthodox Protestant community consists of approximately 250.000 persons; ~1.5% of the Dutch population [15]. Roughly three-quarters of the orthodox Protestants live geographically in the so-called Dutch Bible belt, which stretches from the south-west to the north-east of the Netherlands. The most recent large rubella outbreak among this community occurred in 2004–2005, counting 387 reported cases and 11 cases of CRS [12]. No cases of CRS have been reported since this outbreak [16].

Shortly after the 2004–2005 rubella outbreak in the Netherlands, a small study established a low rubella screening uptake and high rubella seroprevalence among unvaccinated adolescent females in a municipality with a high number of orthodox Protestants [17]. Seroprevalence data from 2016 to 2017 (epidemiology department of the National Institute for Public Health and the Environment) among 137 orthodox Protestant women aged 18-40y showed that 4% (n = 5) were susceptible to rubella; data among 54 orthodox Protestants girls aged 2-17y *showed that* 30% (n = 16) were susceptible to rubella. This indicates a higher susceptibility among the upcoming generation of orthodox Protestant pregnant women. As known from previous studies among orthodox Protestant women, most women want to make an informed and deliberate vaccination decision, with both religious and health-related aspects influencing their vaccination decision [18, 19]. However, more knowledge is needed on orthodox Protestant women’s rubella screening and vaccination intention and its underlying mechanisms. This information can then be used in developing policy to reduce health risks.

A mixed-methods study among unvaccinated orthodox Protestant women was set out using a quantitative approach to determine women’s personal experience, perceived susceptibility for rubella, and their intention of participation in rubella screening and vaccination. Additionally, a qualitative approach was used to explore and understand the underlying mechanisms of women’s perceived susceptibility, and rubella screening and vaccination intention. The ultimate aim of this study is to contribute to the prevention of future CRS cases by informing HCPs and policymakers on how to improve rubella screening and rubella vaccination decision-making support for unvaccinated orthodox Protestant women.

## Methods

In 2017–2019 a cross-sectional online survey study and a semi-structured interview study were conducted among Dutch women who were unvaccinated against rubella, aged 18–40 years, who had an orthodox Protestant background. This mixed-methods study was part of a larger research project on vaccine decision-making on vaccine-preventable diseases during pregnancy among orthodox Protestant women [20].

In the quantitative study, we aimed to include a representative sample of unvaccinated orthodox Protestant women regarding education level, orthodox Protestant church denomination, and residency in an orthodox Protestant municipality. Questionnaires for the survey study were completed between October 2018 and January 2019. Women were recruited to participate through midwife/obstetrical practices, orthodox Protestant (social) media, an orthodox Protestant university of applied science, and key persons (individuals with close contacts in the orthodox Protestant community) in the Netherlands. Means of communication for recruitment were flyers, posters, and online banners referring women to the study’s website with a link to the online questionnaire.

Ten questionnaire items on MMR-vaccination status, age, postal code, level of education, church denomination, relationship status, pregnancy status, and having children were previously used in other studies among the orthodox Protestant community and based on expert knowledge [21, 22]. For this study, we added items on personal experience with rubella, perceived susceptibility for rubella, and intention to participate in rubella screening and rubella vaccination. Women were asked about their personal experience with rubella using five answer categories: I have had rubella myself, one or more of my children have had rubella, in my immediate surroundings someone has had rubella, no one in my immediate surroundings has had rubella, and unknown. Participants who were pregnant and participants with children were asked if they perceived themselves to be susceptible to rubella using the answer categories: not susceptible, susceptible, or I do not know.

In answering the question to score their rubella screening intention, all participants were asked to imagine to be offered screening before a desired pregnancy. Additionally, when next scoring their MMR-vaccination intention, all participants were asked to imagine to be still susceptible to rubella. A four-point Likert scale was used to score participants screening intention and the intention to vaccinate: will certainly not accept, will probably not accept, will probably accept, will certainly accept, or unknown/not applicable.

The semi-structured interviews were conducted between March and August 2017. Participants were recruited using purposeful sampling through key persons and snowball sampling. The interviews were held at the participants’ homes by trained female interviewers (AdM, DvN and WR). To ensure interviews were conducted in a similar way to reduce bias, the first five interviews were conducted by interviewers AdM and DvN together. In addition, the same interview guide was used for all interviews. The topic guide included open-ended questions about personal experience with rubella, personal experience with rubella during pregnancy, perceived susceptibility to rubella during pregnancy, and perceived need for protection against rubella, including vaccination.

In the questionnaire and during the interviews, participants did not receive additional information about rubella, screening and vaccination. Therefore, participants’ answers were based on their basic knowledge on these topics. Interviewees were also invited to complete a questionnaire. Participants’ vaccination status is based on personal report among both interview and survey participants.

### Data analysis

Data analysis started with the descriptive analysis of the quantitative survey data using IBM SPSS Statistics 25. Participants who were vaccinated against rubella, women who did not have an orthodox Protestant background, and women who did not reach the final page of the questionnaire were excluded from analysis. Based on their postal code, participants were classified as living or not living in an orthodox Protestant municipality. Orthodox Protestant municipalities were defined as municipalities with at least 5% votes for the orthodox Protestant political party, the Staatkundig Gereformeerde Partij (SGP) in the Dutch National Elections for seats in the House of Representatives in 2021 [23]. Qualitative data analysis was conducted using the software program ATLAS.ti 9.1.6. Interviews were recorded with a digital voice recorder and transcribed verbatim. Transcripts were analysed using a thematic content analysis approach. Transcripts were coded, and codes were combined into categories. Subsequently, categories were linked to the four main themes of the survey: personal experience with rubella, perceived susceptibility to rubella during pregnancy, intention to accept rubella screening, and rubella vaccination intention.

## Results

One hundred sixty-seven orthodox Protestant women completed the online questionnaire. Among the survey participants, 162 women reported to be unvaccinated against rubella and five women reported an unknown rubella vaccination status. Survey participants were on average 27.3 years old, had a moderate or high education level (56.3% and 36.5%, respectively), 77.8% had a partner or husband, and 65.4% had children and/or was pregnant.(Table 1) 29% (29.3%) was member of a highly conservative church, 59.9% was member of a moderately conservative church, and 10.8% was member of a church with a low level of conservatism. Ten women participated in an interview. All participating women were married and nine women were pregnant and/or had children.(Table 2) The interviewees were member of various orthodox Protestant church denominations.

**Table 1:** Sociodemographic and rubella related variables of unvaccinated orthodox Protestant survey participants (n = 167)

		Mean	Range
**Age** (in years)		27.3	18–40
**Level of education**		**N**	**%**
	Low ^x^	12	7.2%
	Moderate ^+^	94	56.3%
	High ^#^	61	36.5%
**Church denomination**			
	High level of conservatism ^a^	49	29.3%
	Moderate level of conservatism ^b^	100	59.9%
	Low level of conservatism ^c^	18	10.8%
**Living in an orthodox Protestant municipality (n = 164)**			
	Yes, living in a municipality with ≥ 5% votes for SGP*	132	80.5%
	No, living in a municipality with < 5% votes for SGP*	32	19.5%
**Relationship status**			
	Partner/husband	130	77.8%
	No partner	37	22.2%
**Has children and/or is pregnant (n = 162)**			
	Yes	106	65.4%
	No	56	34.6%
**Personal experience with rubella** (multiple responses possible)			
	“Yes, I have had rubella myself”	101	60.5%
	“Yes, my child(ren) has/have had rubella”	9	5.4%
	“Yes, somebody close has had rubella”	40	24.0%
	“No, I do not have any personal experience with rubella”	44	26.3%
**Perceived own rubella susceptibility during pregnancy (n = 110)** *Women who were pregnant and/or with children during survey study*			
	Not susceptible	76	69.1%
	Susceptible	8	7.3%
	I do not know	26	23.6%
**Screening intention (n = 156)**			
	Will certainly refuse screening	36	23.1%
	Will probably not accept screening	47	30.1%
	Will probably accept screening	40	25.6%
	Will certainly accept screening	33	21.2%
**Vaccination intention (n = 146)**			
	Will certainly refuse vaccination	60	41.1%
	Will probably not accept vaccination	59	40.4%
	Will probably accept vaccination	21	14.4%
	Will certainly accept vaccination	6	4.1%

Abbreviation: SGP = Staatkundig Gereformeerde Partij (Reformed Political Party)

x No, primary, prevocational, intermediate secondary or lower vocational, or lower professional education

+ Intermediate vocational education, higher secondary education or pre-university education

# Higher professional education or scientific education

a Reformed Congregations in the Netherlands (GGiN), Old Reformed Congregations (OGG)

b Reformed Congregations (GG) or Restored Reformed Church (HHK)

c Christian Reformed Churches (CGK) or Reformed Bond (within Protestant Church in the Netherlands)

** Voting proportion for the SGP in the Dutch National Elections for seats in the House of Representatives in 2021

**Table 2:** Characteristics of unvaccinated orthodox Protestant interview participants (n = 10)

**Age (in years), range**	23–34
**Church denomination, n**	
High level of conservatism ^a^	4
Moderate level of conservatism ^b^	5
Low level of conservatism ^c^	1
**Living in an orthodox Protestant municipality, n**	
Yes, living in a municipality with ≥ 5% votes for SGP*	6
No, living in a municipality with < 5% votes for SGP*	4
**Relationship status, n**	
Husband	10
No husband	0
**Has children and/or is pregnant, n**	
Yes	9
No	1

Abbreviation: SGP = Staatkundig Gereformeerde Partij (Reformed Political Party)

a Reformed Congregations in the Netherlands (GGiN), Old Reformed Congregations (OGG)

b Reformed Congregations (GG) or Restored Reformed Church (HHK)

c Christian Reformed Churches (CGK) or Reformed Bond (within Protestant Church in the Netherlands)

* Voting proportion for the SGP in the Dutch National Elections for seats in the House of Representatives in 2021

### Personal experience with rubella

Almost three quarters of the participants in the survey study (73.7%) reported a personal experience with rubella. Most experienced rubella themselves (60.5%), or had someone in their direct surroundings who had experienced rubella (24.0%). In the interviews, women recalled having experienced rubella outbreaks in the past but did not consider it as something happening at present. One woman, who thought she might still be susceptible, had been in close contact with ill family members at a birthday party during her pregnancy and could have possibly been infected with rubella.

*“I heard later that it was rubella, so I was infected anyway, so there was really nothing I could have done about it.”* (Interview 6).

### Perceived rubella susceptibility

Among the unvaccinated women who were pregnant and/or had children (n = 110), 76 (69.1%) considered themselves to be not susceptible to rubella, 23.6% were unsure about being susceptible, and eight women (7.3%) considered themselves to be susceptible to rubella. Of the 76 who considered themselves to be not susceptible, 20 women reported they had not had rubella themselves. Of the eight women who considered themselves to be susceptible, two women reported they had had rubella themselves. This indicates that about a quarter of the participants did not know that immunity is acquired by either rubella vaccination or natural infection. In the interviews, five women who did not receive rubella screening (5/8) clarified they were certain they were immune to rubella, because their parents had told them they had had rubella or ‘all of the childhood diseases’ as children.

*“My mother wrote them* (childhood diseases) *all down in a booklet. […] You just got ill and that was part of it, you had measles or you had rubella and then you were happy, then everyone was happy that you had had it, because then you had antibodies.”* (Interview 7)

### Rubella screening intention

More than half of the survey participants (55.8%) were undecided whether they wanted to be screened for rubella susceptibility; 30.1% would probably refuse and 25.6% would probably accept screening. Of the others, 23.1% would certainly refuse and 21.2% would certainly accept screening. Among the interviewees, only two women (2/10) indicated that they had been screened for rubella susceptibility during their pregnancy. Both women were screened at the initiative of their midwife. None of the interviewees had actively requested for screening themselves. Strikingly, some women were uncertain whether their midwife screened them during their pregnancy.

*“I think that’s what you get checked for, at the beginning of the pregnancy. And I think that what came out of it* (the screening), *that I had had that* (rubella).*”* (Interview 1).

### Rubella vaccination intention

Comparable to screening intention, 54.8% of the survey participants were undecided whether they would accept vaccination if they were susceptible; 40.4% would probably refuse, and 14.4% would probably accept vaccination. While 41.1% would certainly refuse rubella vaccination, only 4.1% would certainly accept rubella vaccination. In the interviews, in line with women’s negative attitude towards rubella screening, none of the unvaccinated women would actively request for vaccination. Religious reasons for not doing so were: ‘I trust that God protects us’, ‘God has a purpose for what happens to us in life’, and ‘As a human being you should not want to be in control of the future’. Non-religious reasons were: ‘There is currently no rubella outbreak going on’, ‘It does not feel as an urgent problem which needs to be solved’, and ‘I have had all of the childhood diseases’.

*“If you would often read it is very dangerous to have rubella in your pregnancy. For example: ‘If you are not vaccinated, do it’. Then you would think about it. But not now.”* (Interview 13).

One woman said she would be more motivated to receive vaccination if someone close had have a child affected by a rubella infection during pregnancy. Several interviewees expected they would like to receive information and/or would like to read information about rubella and necessary precautions if there were an rubella outbreak. Especially women who thought they were or might be susceptible to rubella mentioned taking preventive measures if they were pregnant during a rubella outbreak, such as avoiding high-risk locations (primary schools or households with rubella cases).

*“If I were expecting and they had rubella, I wouldn’t go there. Because that can just be really harmful to your baby”.* (Interview 4)

## Discussion

This study provides new information on personal experience with rubella, perceived rubella susceptibility, and rubella screening and vaccination intentions among unvaccinated women of childbearing age in a religious minority group. Most unvaccinated orthodox Protestant women indicated they are familiar with rubella and most women thought they are not susceptible to rubella. However, study results showed that this perceived unsusceptibility is rarely confirmed with laboratory screening. The study showed high indecisiveness and negative attitudes to accept rubella screening or rubella vaccination among survey participants. Qualitative study results revealed religious arguments to object vaccination and women’s perception of absence of imminent threat of rubella, which could partially explain the low screening and rubella vaccination intention.

Concerning women’s perceived susceptibility, the interview outcomes showed that most women rely on their parents’ memory whether they had had rubella as a child. Whether it actually was rubella or a similar childhood disease remains largely unknown as only few interviewees received rubella screening during pregnancy. Evidence that self-reported history of rubella is not always reliable is also shown in a Japanese study among HCPs [24]. Among the unvaccinated HCPs who remembered a history of rubella, 5% did not have rubella antibodies. On the other hand, among unvaccinated HCPs who did not remember having a history of diseases, 62% did have rubella antibodies. In our study, it appeared that not all unvaccinated participants understood that immunity was related to natural infection. A quarter (20/76) of the unvaccinated women who thought they were not susceptible to rubella reported they had not had rubella themselves.

A high degree of the unvaccinated participants was undecided about accepting rubella screening and interviewees showed reluctance to take the initiative to be screened. In Japan, the government provides voluntary-based rubella susceptibility screening and vaccination for adults to eliminate rubella. A study showed that the uptake among women of childbearing age in Japan remains low: only 39% had taken precautionary actions related to rubella prevention [25]. In these women, the main drivers to take action (i.e., checking documented vaccination history, taking rubella antibody screening, or getting vaccinated) were: (1) having knowledge about rubella screening, rubella outbreaks and CRS, (2) having acquaintances who had taken preventive measures, and (3) having a positive attitude towards vaccination [25]. The first driver was found among our interview participants as well. Women mentioned they wanted to gain knowledge on rubella during an outbreak to be able to prevent rubella infection during pregnancy. The second driver is also likely to apply to orthodox Protestants, although it is likely that rubella is a less discussed topic among friends and family members since the last outbreak occurred more than 15 years ago. Moreover, in line with the third driver, orthodox Protestant women’s negative attitude can be partially explained by their indecisiveness or negative intention towards vaccination based on both religious and health-related aspects [18, 19], supplemented by their perception that they had been infected with rubella as a child.

More than half of the survey participants was undecided whether they wanted to receive rubella vaccination. An argument underlying this doubt was women’s perception that rubella is not an imminent problem as they did not regard rubella as a currently common disease. Karafillakis et al. (2017) found that low risk perception of contracting a vaccine-preventable disease is a frequently mentioned concern in vaccine decision-making, that may outweigh perceived benefits to accept vaccination [26].

In a previously conducted study on maternal pertussis vaccination, orthodox Protestant women reported gathering information as an essential need to make a well-considered vaccination decision [18]. In addition to receiving information, women in the maternal pertussis vaccination study wanted sufficient time to search for information themselves, to converse with others about the vaccination, and to deliberate the values they consider to be important concerning the vaccination [18]. It can be assumed that unvaccinated women, when offered rubella screening or vaccination, also need both information and sufficient time to come to a deliberate, informed decision.

The COVID-19 pandemic occurred after data collection for this mixed-methods study. During the pandemic, COVID-19 vaccination uptake was lower in orthodox Protestant municipalities in the Netherlands [27]. This lower uptake was influenced by religious arguments, anti-vaccination sentiments and anti-government sentiments [28]. As these sentiments might also impact the vaccination coverage for other vaccinations, increasing the risk for future infectious disease outbreaks including rubella, it is important to monitor the influence of anti-vaccination sentiments on vaccination uptake in follow-up research.

A strength of this study was the combination of quantitative and qualitative data that enabled us to determine women’s perceived susceptibility, and rubella screening and vaccination intention, and to understand the underlying mechanisms that support their perspectives. The quantitative study sample was found to be representative for the unvaccinated members of the orthodox Protestant community. Consistent with what we know of unvaccinated orthodox Protestants, our participants were also more often member of a moderately or highly conservative church denominations and more often residing in orthodox Protestant municipalities, compared to the overall orthodox Protestant community [15]. Concerning representativeness of education level, we followed the trend of national statistics among Dutch women [29], which revealed that women with a low level of education were underrepresented in our sample. Previous research among the orthodox Protestant community is not conclusive whether education level is associated with vaccination intention and indecisiveness in vaccination intention [21, 22]. Therefore, we cannot interpret if the underrepresentation of respondents with a low level of education led to outcome bias on the intention to accept rubella vaccination. The purposeful sampling method in the qualitative study resulted in a small, yet, varied sample of age, church denomination and residency in orthodox Protestant municipalities. Level of education was not verified with interview participants, therefore it is unknown whether there is sufficient variation regarding education level in this sample. Finally, both the quantitative and qualitative studies were conducted among a specific under-vaccinated group, namely Dutch orthodox Protestant women, thereby diminishing the applicability of the results to other under-vaccinated groups.

## Conclusion and recommendations

This study indicates that half of the unvaccinated orthodox Protestant study participants is undecided whether or not to accept rubella screening and vaccination. This indecisiveness is likely to be related to women’s unconfirmed assumptions that they are not susceptible to rubella and their perceived low risk of contracting rubella due to the absence of an outbreak. To prevent CRS cases in future rubella outbreaks, several recommendations can be made. Firstly, unvaccinated pregnant women and women of childbearing age should be made aware by e.g. national and regional public health institutes that they possibly are susceptible to rubella, which puts their unborn child at risk for CRS. In light of an imminent rubella outbreak in the Netherlands, a tailored education campaign should be prepared aimed at and established in cooperation with the under-vaccinated orthodox Protestant community, as they are most at risk of rubella infection. Secondly, Dutch HCPs involved in (pre)pregnancy care should be reminded to follow guidelines recommending rubella screening to unvaccinated women in order to assess their rubella susceptibility status. HCPs should explain the added value of laboratory screening if women think they have had rubella as child, as rubella can be mistaken for another childhood disease that causes a rash. Thirdly, all HCPs involved in the care of these women should note that they should support these women in their decision-making to enable them to make their own deliberate and informed decision on rubella screening and, if necessary, subsequent vaccination.

## Data Availability

The dataset used and/or analysed during the current survey study are available from the corresponding author (JH) on reasonable request. The datasets generated and/or analysed during the current interview study are not publicly available as the raw interviews and transcripts contain sensitive information and study participants are part of a close-knit community, even anonymized raw data can seriously compromise their confidentiality, but are available from the corresponding author on reasonable request.

## Abbreviations

CRS: congenital rubella syndrome
HCP: health care provider
MMR: measles-mumps-rubella (vaccination)
SGP: Staatkundig Gereformeerde Partij (Reformed Political Party)
